# Africa's 32 Cents Solution for HIV/AIDS

**DOI:** 10.1371/journal.pntd.0000430

**Published:** 2009-05-26

**Authors:** Peter J. Hotez, Alan Fenwick, Eyrun F. Kjetland

**Affiliations:** 1 Sabin Vaccine Institute, Washington, D.C., United States of America; 2 Department of Microbiology, Immunology, and Tropical Medicine, The George Washington University, Washington, D.C., United States of America; 3 Schistosomiasis Control Initiative, Imperial College London, London, United Kingdom; 4 Centre for Imported and Tropical Diseases, Department of Infectious Diseases Ullevaal, Oslo University Hospital, Oslo, Norway; 5 University of Oslo, Oslo, Norway

*By preventing urogenital schistosomiasis in sexually active females through simple and low-cost methods, we have an innovative and timely opportunity to reduce and possibly interrupt HIV/AIDS transmission throughout many rural areas of sub-Saharan Africa.*

More than 90% of the world's 207 million cases of schistosomiasis occur in sub-Saharan Africa [1], making this condition (as well as hookworm infection) one of the most common neglected tropical diseases in the region [1],[2]. Based on additional information that schistosomiasis causes chronic anemia and inflammation associated with severe disability among children, adolescents, and young adults, the disease burden resulting from schistosome infections in Africa may actually rival better known conditions such as HIV/AIDS, tuberculosis, and malaria [1],[3].

**Figure 1:**
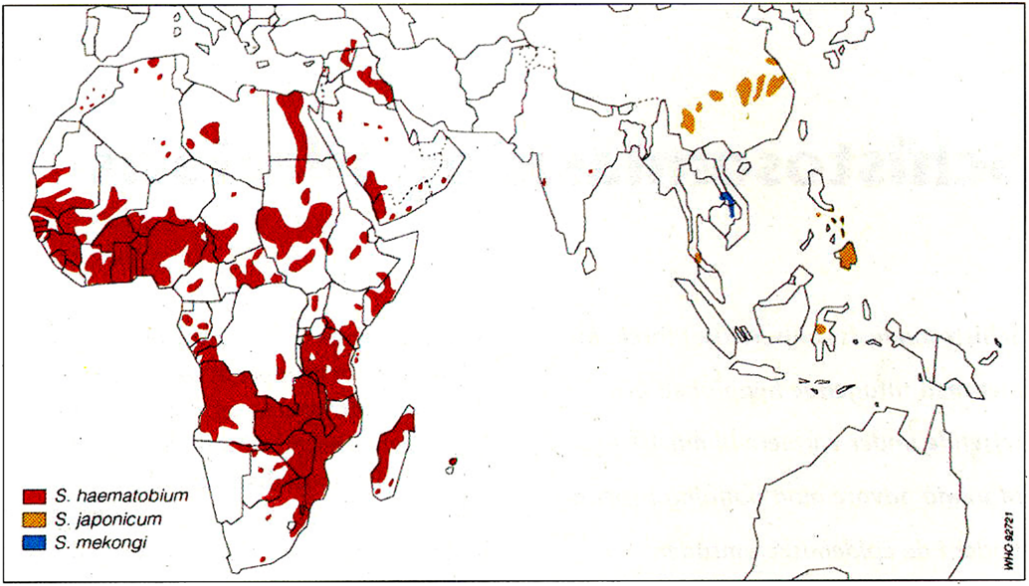
Global distribution of *S. haematobium* Infection in Africa (As Well As *S. japonicum* and *S. mekongi* Infections in Asia) from the World Health Organization. Reprinted with the permission of the WHO (from page 16 in [41], image WHO 92721).

Approximately two-thirds of the cases of schistosomiasis in sub-Saharan Africa result from urinary tract infections caused by *Schistosoma haematobium*. Shown in Figure 1 is a map of the geographic distribution of urinary schistosomiasis in Africa, in which the major foci of infection occur in southeastern Africa, i.e., Kenya, Malawi, Mozambique, Tanzania, Zimbabwe, South Africa, and Zambia; in West Africa, i.e., Burkina Faso, Mali, Niger, and Nigeria; and in Angola and Sudan. However, urinary schistosomiasis has been almost eliminated from Egypt [4]. Of the estimated 112 million cases of *S. haematobium* infection in sub-Saharan Africa, approximately 70 million are associated with hematuria, 18 million with major bladder wall pathology, and 9.6 million with major hydronephrosis leading to severe kidney damage [5]. The results from Egypt suggest that these consequences may be avoidable provided that regular praziquantel treatment reaches school-age children at risk.

In addition, there is yet another and largely hidden condition associated with chronic *S. haematobium* infection. Studies from the *S. haematobium*–endemic areas of sub-Saharan Africa indicate that between 33% and 75% of infected women also suffer from female genital schistosomiasis (FGS) of the lower genital tract [6]. FGS results from deposition of the schistosome eggs in the uterus, cervix, vagina, or vulva, and the ensuing host inflammation is comprised of granulomas, fibrosis, and angiogenesis (pathological localized blood vessel formation) [6],[7]. The cervix and the vagina are the most common sites of egg deposition, although post-mortem studies have revealed that the upper genital tract is also frequently involved [8]. FGS was identified as an established clinical feature of *S. haematobium* infection in Africa beginning in the 1950s and early 1970s [8],[9], but it is only within the last two decades that its full clinical spectrum has been revealed [6], [7], [10]–[12]. The pathognomonic lesions associated with *S. haematobium* infection in the female genitals are mucosal grainy sandy patches linked to egg granulomas, which are usually associated with mucosal bleeding (especially “contact bleeding” during pelvic examination or sexual intercourse) and abnormal blood vessels [7]. The homogenous yellow sandy patches of FGS may mimic the sexually transmitted diseases (STDs) and have been found to be associated with a variety of important clinical manifestations including bleeding disorders, dyspareunia, pelvic and lower abdominal pain, vaginal discharge, pollakisuria, stress incontinence, and infertility [6],[7],[11],[12].

Now there is new evidence indicating that the sandy patches of FGS increase susceptibility to HIV/AIDS [13]–[15]. Schistosomiasis occurs predominantly in rural areas, and although HIV/AIDS is often considered an urban or semi-urban infection, there are also important and emerging foci of epidemic AIDS in African rural communities [16]. A cross-sectional study from a rural Zimbabwean community reveals that women aged 20 to 49 with FGS exhibit a 3-fold risk of having HIV relative to women without FGS [14]. Presumably, the schistosome egg granulomas produce genital lesions and mucosal barrier breakdown to facilitate HIV viral entry in a manner similar to that described for STDs such as viral infection from herpes simplex virus 2 or human papillomavirus, or syphilis [6], [16]–[19]. Alternatively, contact bleeding could promote direct entry of HIV into the bloodstream, or the CD4+ cells present in schistosome egg granulomas could provide a nidus for HIV viral entry and replication [6].

Regardless of the mechanism, it is important to establish whether anti-schistosomal treatment could represent an innovative HIV/AIDS prevention strategy. Towards this end, praziquantel is both a highly effective and low-cost anti-schistosomal therapy and a prophylactic agent against schistosomal morbidity in adults. The London-based Schistosomiasis Control Initiative (SCI) is currently working with ministries of health to conduct appropriate national control programs for schistosomiasis and other helminth infections [20]. In Burkina Faso, SCI has shown that when provided as a single mass treatment in Burkina Faso praziquantel can reduce the prevalence of *S. haematobium* infection by 84% among girls and 78% overall for at least 2 years [21]–[22]. SCI has also demonstrated similar successes in the other seven sub-Saharan African countries where it operates [20]. SCI is currently developing country-specific strategies based on annual or biannual treatment of school-age children according to collected epidemiological evidence of prevalence and intensity of infections, and based on the World Health Organization (WHO) recommendations [23]. The WHO strategy is of mass drug administration (MDA) with praziquantel for all school-age children (enrolled and not enrolled) once every 2 years in moderate risk communities where the prevalence of schistosomiasis exceeds 10%, and annually where the prevalence exceeds 50% [24]. Among women with FGS, praziquantel will result in parasitological cure and halt the appearance of new ova in the urine and presumably in the genital tract [25]. However, while there are instances when treatment will also reverse some of the lower genital tract abnormalities associated with FGS [26], regrettably praziquantel anthelminthic therapy among adult women (with established genital schistosomiasis) does not alleviate genital lesions, contact bleeding, or vessel abnormalities [25]. Therefore, based on the presumed mechanism by which FGS promotes HIV infection, it is unlikely that praziquantel treatment of affected adults will interrupt the horizontal transmission of HIV/AIDS.

**Figure 2:**
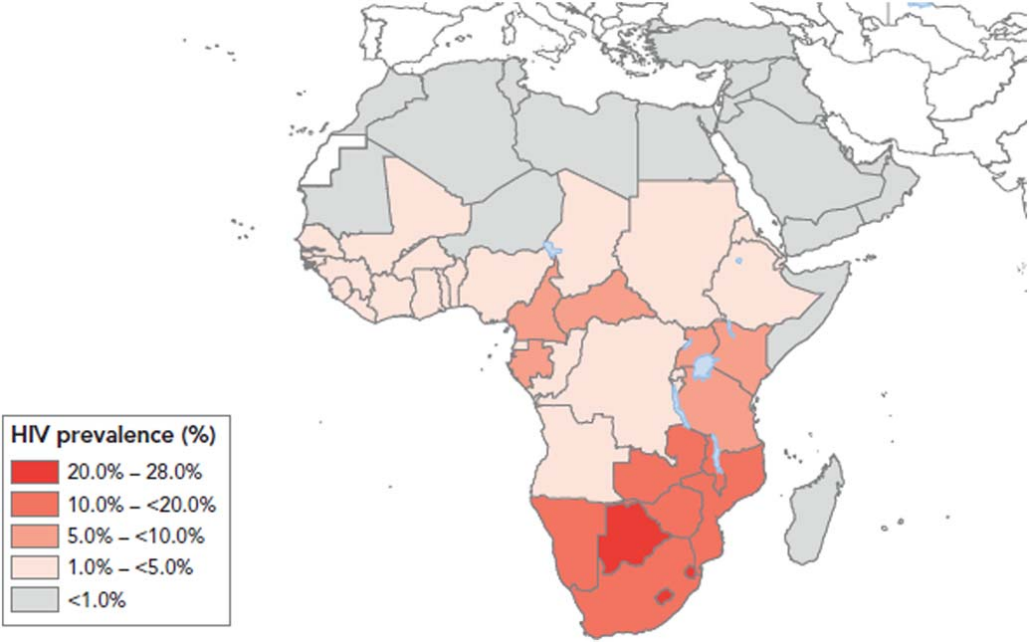
Global Distribution of HIV/AIDS in Africa, Showing the HIV Prevalence (%) in Adults (15–49) in Africa, 2007. Reprinted with the permission of UNAIDS (from page 39 in [30], Figure 2.8).

Our hypothesis is that treatment of younger females at risk of schistosomiasis is therefore critical, and that praziquantel treatment of young school-age girls will prevent the development of FGS and therefore represent an innovative AIDS prevention strategy in areas of HIV and *S. haematobium* transmission. In a cross-sectional study in northern Zimbabwe, women who report having received praziquantel treatment before the age of 20 exhibited an absence of sandy patches and contact bleeding that was independent of adult waterbody contact [27]. Based on this study, we believe there is an urgent need to demonstrate the impact of praziquantel on HIV transmission in prospective studies among school-age girls living in HIV/AIDS and schistosomiasis co-endemic areas, such as in the southeastern countries of Malawi, Mozambique, Tanzania, and Zimbabwe, areas which exhibit high prevalences of both *S. haematobium* and HIV infections (Figures 1 and 2). We further support efforts to seek additional funds in order to investigate this innovative HIV/AIDS control tool. There is also some evidence that *Schistosoma mansoni* infection could result in FGS, although at a lower rate than *S. haematobium* infection [28]; this also warrants further study, particularly given that vaccines against both *S. haematobium* and *S. mansoni* infection are under development [2],[29].

However, we also feel that the evidence for FGS promoting HIV transmission is sufficiently compelling that it would be unethical to withhold mass treatment for many years while awaiting the results of a prospective study that follows school-age girls as they become sexually active women (and exposed to HIV). Van der Werf et al. estimate there are 56 million African school-age children with *S. haematobium* infection [5], and presumably 28 million *S. haematobium*–infected school-age girls. There are also another 14 million preschool children and 7 million infected preschool girls in Africa (Table 1). Based on the estimate that between 33% and 75% of women infected with *S. haematobium* also exhibit signs and symptoms of FGS [6], and taking the value of 54% as the mean of these ranges, it follows that approximately 19 million girls could develop FGS over the next decade in sub-Saharan Africa. With an appropriate school-based praziquantel treatment regime (annually in highly endemic areas), it would be possible to prevent 16 million cases of FGS in sub-Saharan Africa through this approach. This assumes that bi-annual praziquantel treatments can reduce the prevalence of schistosomiasis by 84% as shown previously in Burkina Faso [21]. Although outside of South Africa there are not well-established incidence figures for HIV/AIDS in the region, the Joint United Nations Programme on HIV/AIDS (UNAIDS) reports that 1.9 million new cases of HIV/AIDS occurred in sub-Saharan Africa in 2007 [30], representing 0.25% of the total population of 770 million in the region [31]. By extrapolation we can estimate that among a cohort of 16 million women with FGS having a 3-fold increased risk of HIV/AIDS (representing 0.75% of this population), we could potentially prevent up to 120,000 new cases of HIV/AIDS through regular praziquantel treatments in the next decade. Almost certainly, this figure is a conservative estimate. In addition, schistosomiasis is known to cause hematospermia and increased levels of immunological activity in sperm (including increased leukocytes and elevated cytokine levels), decreasing after treatment [32],[33]. For anatomical reasons, male genital schistosomiasis does not appear to make men more susceptible to HIV/AIDS. However, dually infected men have been hypothesized to increase the risk of HIV/AIDS transmission to women through higher HIV viral loads in seminal ejaculate [33]–35. The viral loads in semen of schistosomiasis-infected males has not yet been investigated.

**Table 1:** School-Age and Preschool Children with *S. haematobium* Infection and FGS (Based on Data in [4],[20],[28]).

Preschool Children Infected with *S. haematobium*	School-Age Children Infected with *S. haematobium*	Preschool and School-Age Children (and Girls) with *S. haematobium*	Preschool and School-Age Girls That Will Develop FGS (Calculated at a Rate of 54%) over the Next Decade	Conservative Estimate of Cases of FGS That Could Be Prevented with a Single Round of Praziquantel (Based on a Reduction Rate of 84% Prevalence [20])	Costs of Annual Treatment of 70 Million Preschool and School-Age Children Infected with *S. haematobium* (at a Cost of $0.32 per Child Based on [28])	Costs of Bi-Annual Treatment For 10 Years
14 million children	56 million children	70 million children and 35 million girls	19 million girls	16 million girls	$22.4 million	$112 million

MDA of praziquantel is both low cost and cost efficient. The SCI has determined that even considering all of the delivery costs, the mass treatment program of school-age children in Burkina Faso was conducted for only $0.32 per child [36]. Therefore, 70 million children infected with *S. haematobium* could be treated for $22 million, which, if repeated bi-annually for 10 years, would cost approximately $112 million (Table 1). By comparison, PEPFAR (the US President's Emergency Plan for AIDS Relief) is projected to spend $18.8 billion over the next 5 years (representing the largest international health initiative ever devoted to a single disease), including $1.34 billion annually in support of treatment programs and $601 million annually in support of treatment strategies [37]. Therefore, periodic praziquantel administration would represent a very small add-on cost to the PEPFAR budget, and yet would have the dual benefits of making dramatic improvements in the reproductive health of women living in rural Africa, likely reducing HIV/AIDS transmission. Shown in Table 2 are the prevalence rates of both HIV/AIDS and schistosomiasis in the 15 countries where PEPFAR operates. At the minimum, we strongly advocate for the introduction of low-cost praziquantel MDA in the PEPFAR countries of Mozambique, Tanzania, and Zambia by using PEPFAR funds in support of SCI activities through a Global Network for NTDs [38]–[40]. This represents a $0.32 solution that could have enormous benefits for young African women and a huge potential beneficial impact on Africa's AIDS epidemic.

**Table 2:** HIV/AIDS and Schistosomiasis Prevalence Rates in the 15 PEPFAR Countries (Based on Information in [1] and [42]).

Country	HIV/AIDS Prevalence in Adults (15–49) in 2007 (UNAIDS)	Women (15+) Infected with HIV/AIDS in 2007	Number of Cases of Schistosomiasis (Prevalence)	Population at Risk for Schistosomiasis
Botswana	23.9%	170,000	0.2 million (10%)	1.8 million
Cote d'Ivoire	3.9%	250,000	6.7 million (40%)	16.7 million
Ethiopia	2.1%	530,000	5.0 million (7%)	37.5 million
Guyana	2.5%	7,100	None	None
Haiti	2.2%	58,000	None	None
Kenya	ND	ND	7.4 million (23%)	32 million
Mozambique	12.5%	810,000	13.2 million (70%)	18.9 million
Namibia	15.3%	110,000	<0.1 million (1%)	0.2 million
Nigeria	3.1%	1,400,000	29 million (23%)	112.8 million
Rwanda	2.8%	78,000	0.5 million (6%)	5.0 million
South Africa	18.1%	3,200,000	4.9 million (11%)	27.9 million
Tanzania	6.2%	760,000	19.0 million (51%)	37 million
Uganda^a^	5.4%	480,000	5.3 million (20%)^1^	16.7 million^1^
Vietnam	0.5%	76,000	None	None
Zambia	15.2%	560,000	2.9 million (27%)	10.8 million

a All of the schistosomiasis in Uganda is caused by *S. mansoni,* which may also affect the genitals according to case reports. However, the prevalence and severity of gynecological *S. mansoni* morbidity are unexplored.
